# Supplementary material for: A systematic benchmark of high-accuracy PacBio long-read RNA sequencing for transcript-level quantification
Source: Genome Biol. 2026 Feb 25;27:110. doi: 10.1186/s13059-026-03988-1 (PMC13040695; doi:10.1186/s13059-026-03988-1)
Supplement: Supplementary file 1 — Additional file 1: Fig. S1. Quality control of all samples in our dataset highlights quality issues in Day2-1 for Kinnex. Fig. S2. Biological exploration on the iPSC-EC differentiation dataset reveals differential splicing, differential expression and a moderate amount of novel isoforms. Fig. S3. Performance of Kinnex relative to Illumina on DTE, and relative and absolute quantification, including when downsampled to approximately base-normalized depth. [file 13059_2026_3988_MOESM1_ESM.pdf]

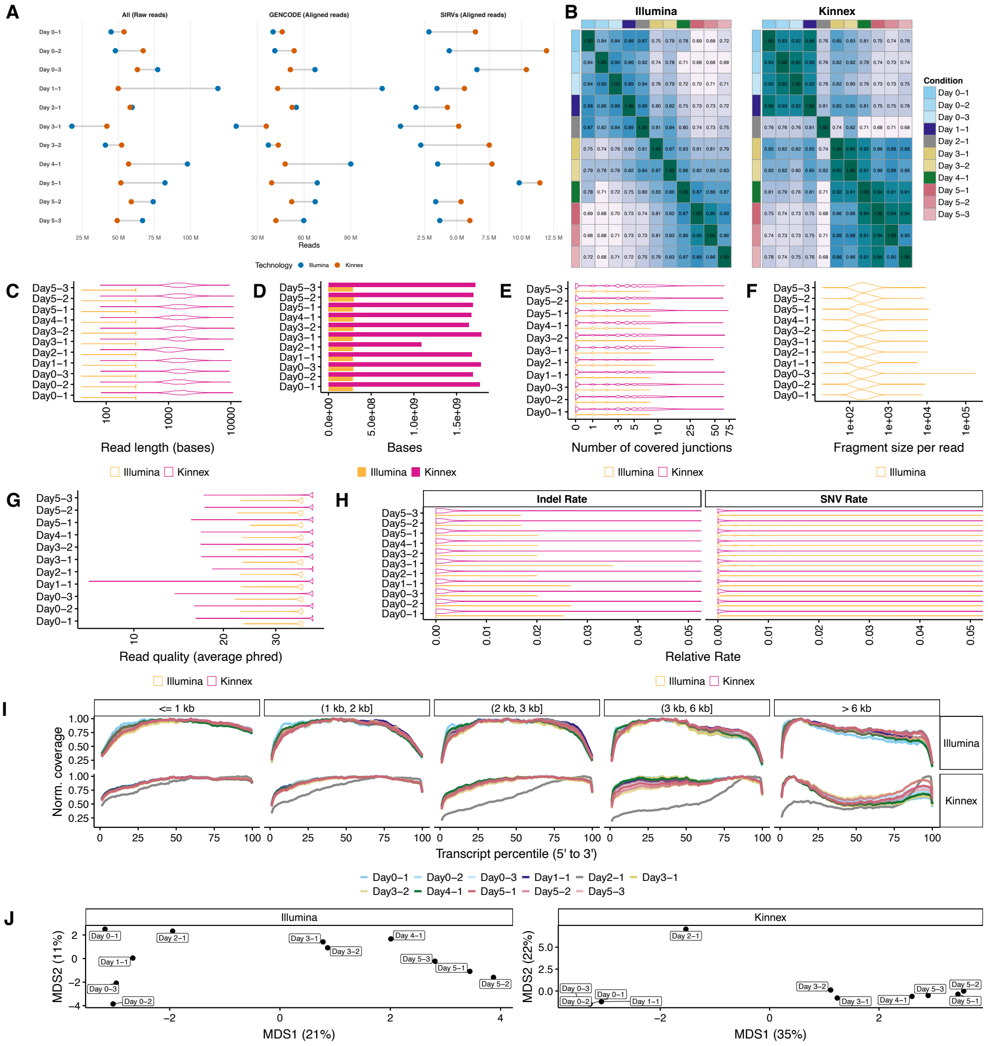
A
All (Raw reads)
GENCODE (Aligned reads)
SIRVs (Aligned reads)
Reads
Technology Illumina Kinnex
B
Illumina
Kinnex
Condition
Day 0–1
Day 0–2
Day 0–3
Day 1–1
Day 2–1
Day 3–1
Day 3–2
Day 4–1
Day 5–1
Day 5–2
Day 5–3
C
Read length (bases)
Illumina Kinnex
D
Bases
Illumina Kinnex
E
Number of covered junctions
Illumina Kinnex
F
Fragment size per read
Illumina
G
Read quality (average phred)
Illumina Kinnex
H
Indel Rate
SNV Rate
Relative Rate
Illumina Kinnex
I
<= 1 kb
(1 kb, 2 kb]
(2 kb, 3 kb]
(3 kb, 6 kb]
> 6 kb
Illumina
Kinnex
Norm. coverage
Transcript percentile (5' to 3')
J
Illumina
Kinnex
MDS1 (21%)
MDS2 (11%)
MDS1 (35%)
MDS2 (22%)

Fig. S1: Quality control of all samples in our dataset highlights quality issues in Day2-1 for Kinnex. A. Number of raw and aligned reads per technology, stratified by their source across all samples. **B.** Spearman correlation heatmap of transcript-level quantification across all samples, highlighting sample-similarities across the full differentiation for each technology and quality issues with Day 2-1 for Kinnex. **C.** Read lengths of 1M randomly sampled reads aligning to the human genome, stratified by technology for all samples, highlighting quality issues with Day2-1 for Kinnex. For Illumina, the length was calculated across both ends. **D.** Number of base pairs sequenced from 1M randomly sampled reads collected for each technology (same reads as **C**). **E.** Number of covered junctions per read from 1M randomly sampled reads collected for each technology (same reads as **C**). **F.** Fragment size distribution of 1M randomly sampled reads collected for each technology (same reads as **C**). **G.** Average base quality of 1M randomly sampled reads aligning to the human genome, stratified by technology for all days (same reads as **C**). Average base quality for Illumina was determined from both ends. **H.** Average edit distance per read, stratified by indel vs SNV errors, of 1M randomly sampled reads collected for each technology (same reads as **C**). **I.** Normalized coverage of reads aligning to 2,500 randomly sampled GENCODE transcripts each across gene body percentiles, stratified by technology and transcript length, across all samples, highlighting quality issues with Kinnex Day2-1. **J.** Transcript-level quantification-based MDS plots highlighting quality issues for Day2-1 for Kinnex, stratified by technology.

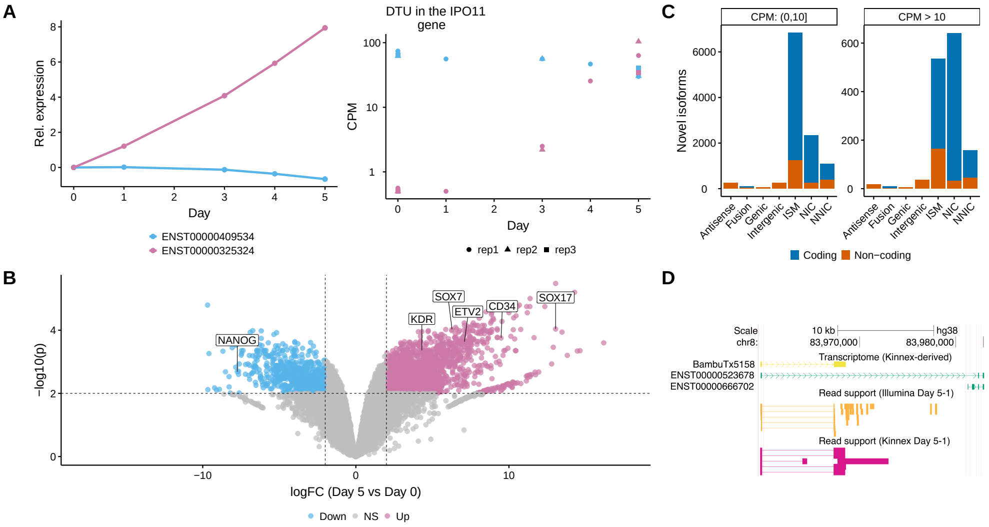

Fig. S2: **Biological exploration on the iPSC-EC differentiation dataset reveals differential splicing, differential expression and a moderate amount of novel isoforms. A.** Estimated relative (left) and absolute observed (right) expression of two transcripts belonging to the *IPO11* gene across the differentiation. Relative expression estimated using a cubic spline with two degrees of freedom (see **Methods**). **B.** Volcano plot of Differential Gene Expression between Day 0 and Day 5, highlighting genes specific to pluripotency (light blue) and primordial endothelial cells (pink). **C.** Number of novel transcripts in each category as annotated by SQANTI3, stratified by mean CPM in the two samples with the highest CPM for a particular transcript, colored by coding status as predicted by ORFanage. **D.** Browser track highlighting an example of a relatively confidently identified novel transcript that has read support in both Kinnex and Illumina data.

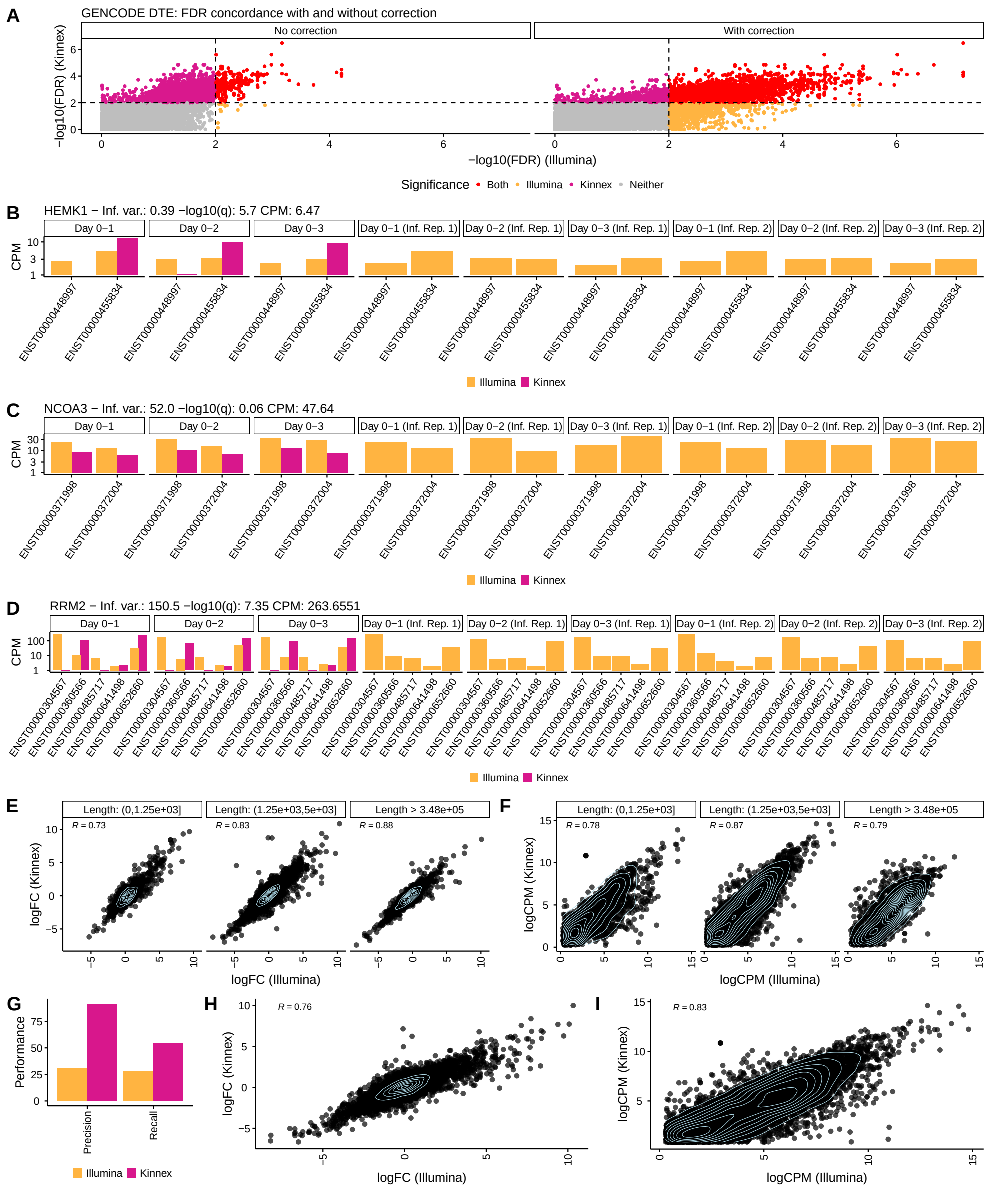

A
GENCODE DTE: FDR concordance with and without correction
No correction
With correction
−log10(FDR) (Kinnex)
−log10(FDR) (Illumina)
Significance • Both • Illumina • Kinnex • Neither
B
HEMK1 − Inf. var.: 0.39 −log10(q): 5.7 CPM: 6.47
C
NCOA3 − Inf. var.: 52.0 −log10(q): 0.06 CPM: 47.64
D
RRM2 − Inf. var.: 150.5 −log10(q): 7.35 CPM: 263.6551
Day 0−1
Day 0−2
Day 0−3
Day 0−1 (Inf. Rep. 1)
Day 0−2 (Inf. Rep. 1)
Day 0−3 (Inf. Rep. 1)
Day 0−1 (Inf. Rep. 2)
Day 0−2 (Inf. Rep. 2)
Day 0−3 (Inf. Rep. 2)
CPM
Illumina Kinnex
E
F
Length: (0,1.25e+03]
Length: (1.25e+03,5e+03]
Length > 3.48e+05
logFC (Kinnex)
logFC (Illumina)
logCPM (Kinnex)
logCPM (Illumina)
G
Performance
Precision
Recall
H
I

Fig. S3: **Performance of Kinnex relative to Illumina on DTE, and relative and absolute quantification, including when downsampled to approximately base-normalized depth. A.** Concordance in per-transcript DTE q-values between Illumina and Kinnex with and without applying an inferential variability correction for Illumina (see **Methods**). Only transcripts that were not filtered for all technologies (Kinnex, Illumina without correction, Illumina with correction) are shown. **B.** Exemplary quantification of the three Day 0 replicates for Illumina and Kinnex and two inferential Day 0 replicates for Illumina on the *HEMK1* gene. **C.** Exemplary quantification of the three Day 0 replicates for Illumina and Kinnex and two inferential Day 0 replicates for Illumina on the *NCOA3* gene. **D.** Exemplary quantification of the three Day 0 replicates for Illumina and Kinnex and two inferential Day 0 replicates for Illumina on the *RRM2* gene. **E.** Concordance of the first replicate per-transcript log-fold changes between Kinnex and Illumina stratified by length in nts. **F.** Concordance of the first replicate of Day 0 per-transcript log CPM values between Kinnex and Illumina, stratified by length in nts. For fairness, in panels E-F, both technologies were downsampled to 30M reads. **G.** Precision and recall of de-novo transcript discovery on three Day 0 (E1 mix) SIRV replicates by technology, when run on approximately base-normalized data. Illumina was run on a downsampled read set consisting of 2.5 M reads, while Kinnex was run on a downsampled read set of 0.25 M reads. **H.** Concordance of the first replicate per-transcript log-fold changes between Kinnex and Illumina when run on approximately base-normalized data. Illumina was run on 30 M downsampled reads, while Kinnex was run on 5 M. **I.** Concordance of the first replicate per-transcript log CPM values between Kinnex and Illumina. Illumina was run on 30 M downsampled reads, while Kinnex was run on 5 M.
